# The rise of new approach methodologies (NAMs) for environmental safety assessment of chemicals: A systematic review of the current status, challenges and strategies for broader adoption of NAMs

**DOI:** 10.1016/j.namjnl.2026.100115

**Published:** 2026-07-15

**Authors:** Maria Christou, Anastasia Georgantzopoulou, Maria Hultman, You Song, Aina Charlotte Wennberg, Sophie Mentzel, Michelle Embry, Amelie Ott, Takahiro Suzuki, Giacomo Grassi, Kyle Roush, Adam Lillicrap

**Affiliations:** a Norwegian Institute for Water Research (NIVA), Oslo, Norway; b Health and Environmental Sciences Institute, Washington, DC, USA; c International Collaboration on Cosmetics Safety (ICCS), NY, USA; d Kao Corporation, R&D Safety Science Research, Tochigi, 321-3497, Japan; e L’Oréal, Paris, France; f Procter & Gamble, Cincinnati, OH, USA

**Keywords:** 3Rs, NAMs, Systematic literature review, Animal alternatives

## Abstract

Traditional ecotoxicity testing relies heavily on vertebrate animal models, which are resource-intensive, low-throughput, and raise ethical concerns under the principle of the 3Rs (Reduction, Refinement, Replacement). New Approach Methodologies (NAMs), including *in silico, in chemico, in vitro* and *ex vivo*, offer promising alternatives for environmental chemical hazard assessment. While NAMs have gained traction in human health risk assessment, their regulatory acceptance for environmental safety remains limited due to technical, scientific and policy barriers. This paper presents an overview of a literature review related to the status, challenges and future strategies for broader adoption of NAMs for environmental safety assessment of chemicals. The review´s objectives were to: (1) systematically review the current state of NAMs for aquatic toxicity assessment, (2) identify gaps in NAM readiness and endpoint coverage, and (3) provide recommendations to accelerate regulatory acceptance and integration of NAMs into environmental safety frameworks. A systematic literature review was conducted using PubMed followed by the SWIFT-Active Screener tool, focusing on NAMs applicable to aquatic organisms (fish, amphibians, marine mammals). The review excluded terrestrial and invertebrate models. Search terms considered within the scope of the review included *in silico, in chemico, in vitro* and *ex vivo*. While embryo assays may not be considered a NAM by all geographical regions and stakeholders, information from existing embryo assays was also reviewed to reduce the reliance on protected vertebrate life stages. The screening identified 4275 unique articles and 1310 were selected for full-text review. A database containing all the reviewed information is freely available and contains 18 metadata fields including for example the captured assay type, species, endpoints, and readiness level. This review provides a comprehensive roadmap for transitioning toward animal-free environmental safety assessment. By addressing gaps in readiness, standardization, and mechanistic integration, NAMs can become central to next-generation risk assessment, supporting global initiatives to phase out vertebrate testing.

## Graphical abstract

**Figure:**
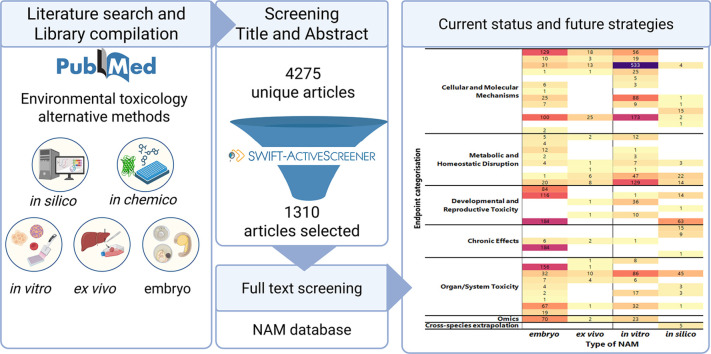

## 1 Introduction

The use of laboratory animals for toxicity testing is highly cost-inefficient, resource-intensive, low-throughput and counter to animal welfare considerations and the principles of the 3Rs (*i.e.*, reduction, refinement and replacement as first defined by Russell and Burch (1959)). New Approach Methodologies (NAMs) offer promising solutions to avoid the need for *in vivo* animal tests for assessing the effects of chemicals on the environment. NAMs may also prove to be more informative than current ecotoxicity test guidelines that support hazard identification and mechanistic understanding. NAMs include any predictive *in silico, in chemico, in vitro* and *ex vivo* methods that can be used to perform environmental safety assessments. NAMs are not necessarily new methods, but the possibilities of inclusion in regulation, and their use for replacement of *in vivo* methods, may be new. Many activities and national work plans have been proposed worldwide to facilitate the implementation of NAMs. In the United States, the Environmental Protection Agency´s Office of Chemical Safety and Pollution Prevention and Office of Research and Development published a NAM Work Plan and the Interagency Coordinating Committee on the Validation of Alternative Methods (ICCVAM) strategic roadmap. In Canada, the Pest Management Regulatory Agency’s 2016–2021 strategic plan and the strategy to replace, reduce or refine vertebrate animal testing from Health Canda and Environment and Climate Change Canada were published. In the European Union, several initiatives such as Directive 2010/63/EU and the EU Chemicals Strategy for Sustainability (CSS), Towards a Toxic-Free Environment, have been established. Additionally, the European Commission roadmap towards phasing out animal testing was recently published in June 2026. This roadmap initiative stems from the European Citizens' Initiative "Save Cruelty-Free Cosmetics – Commit to a Europe Without Animal Testing" and a subsequent Commission communication outlining the intention to support and accelerate the path to implementing NAMs. Common to all is the adoption of NAMs to ensure protection of human health and the environment (for detailed information please see review by Stucki et al. (2022)). Many of these activities targeting the shift to using NAMs have historically received both public and industry support as reflected in the long-standing cosmetic industry commitment to not conduct tests on animals.

Although several NAMs have already been validated and accepted for human chemical safety assessments, there are only a few which have been validated and accepted for environmental chemical hazard assessments. Examples of these environmentally focused NAMs that have been published as internationally accepted test guidelines within the OECD test guideline series include OECD 236 and 249 (Fish Embryo Toxicity (FET) test and Rainbow trout-gill cytotoxicity assay) (OECD, 2013a, 2021a). The RTgill-W1 cytotoxicity assay and the FET test are generally similarly sensitive to a broad range of chemicals (Belanger et al., 2013; Tanneberger et al., 2013) and show a good correlation with *in vivo* acute fish toxicity (AFT) data. However, some chemicals, such as allyl alcohol, show a weaker correlation to *in vivo* AFT data (Knöbel et al., 2012). As such, the use of these assays as a standalone replacement to the AFT is currently limited by regulatory authorities due to the perceived lack of sensitivity to substances which require biotransformation or cause neurotoxicity. However, they can be used in a weight of evidence approach (*e.g.* Lillicrap et al., 2020 and Moe et al., 2020).

Other examples of established NAMs are *in silico* methods such as Quantitative Structure Activity Relationships (QSARs), which are used by some regulators and risk assessors for screening the effects of chemicals in the environment. Macmillan et al. (2025) have developed a defined approach integrating data from three freely available (Q)SARs with data from the *in vitro* RTgill-W1 assay to provide acute aquatic hazard classifications.

In addition to well-established NAM approaches, a suite of novel tools is being developed that allow for incorporation of additional hypothesis-driven, exposure-based lines of evidence. High-throughput screening assays, advanced *in vitro* models (including 3D cultures and micro-physiological systems) and omics are promising tools that can support fast and mechanism-driven hazard assessment. The study of omics involves biological responses at the transcriptome, proteome, metabolome and lipidome level as an early indicator of toxicity at sublethal concentrations. By integrating multiple omics (multi-omics), complex networks from genes to phenotypes and sub-cellular responses can be efficiently captured, revealing insights into sequential toxic mechanisms and adverse outcomes. While these novel tools represent a significant shift in capability and understanding of chemical-induced hazard potential, their use in regulatory settings is currently limited. This may be due to lack of standardisation, an unclear link to adverse outcomes, the high variability depending on tissue type or life stage or the fact that there aren’t enough well-validated biomarkers for use in regulatory decision making.

As chemicals can potentially produce diverse types of biological effects, there is an urgent need to assemble a battery of NAMs with a broad coverage of different modes of action for assessing chemical hazards to the aquatic environment. This review was aimed at thoroughly assessing the current state of the science on available NAMs and their potential applications for assessing aquatic hazards of chemicals in the environment. The main objectives were to:

1. Perform a systematic review on the current state of the art of ecotoxicity related NAMs for the future development of an environmental safety toolbox.
2. Identify gaps in NAM readiness for possible future research needs.
3. Provide key recommendations for further development of NAMs and means to build confidence in the broader use of NAMs.

For the purposes of this paper, the NAMs included *in silico* approaches (*i.e.*, any computerised prediction), *in chemico* methods (*e.g.*, knowledge of chemical reactivity to provide information relating to toxicity), *ex vivo* procedures (*i.e.*, method isolating an organ or tissue from a living organism to study effects outside the organism), *in vitro* assays (*i.e.*, cells cultivated in an artificial environment in well plates or petri dishes outside of the organism) and embryo assays (*i.e.*, use of non-protected early life stage test organisms pre larval development). While some regions and organizations do not consider embryo assays as NAMs, this review included those assays to benefit from existing data having been generated with these approaches. *Ex vivo* data was considered similarly and included to extract value of existing data, though sourcing of the organs or tissues need to be considered for utilization in a NAM context. Anything *in vivo* herein was considered to reflect a test with a whole organism such as the fish acute toxicity test (*e.g.* OECD 203, OECD, 2019).

## 2 Methodological approach for systematic review

### 2.1 Systematic literature search strategy

The review was limited to evaluating the status of NAMs that are applicable for assessing the environmental safety of chemicals but did not include terrestrial or invertebrate-based ecotoxicity methods as these areas were considered outside of the scope of the project. In addition, alternative approaches for assessing chemicals causing endocrine disruption as an endpoint were not the focus of this study as these have previously been reviewed extensively by Mitchell et al. (2023) but were nevertheless registered in the database of aquatic toxicity testing.

**Fig. 1:**
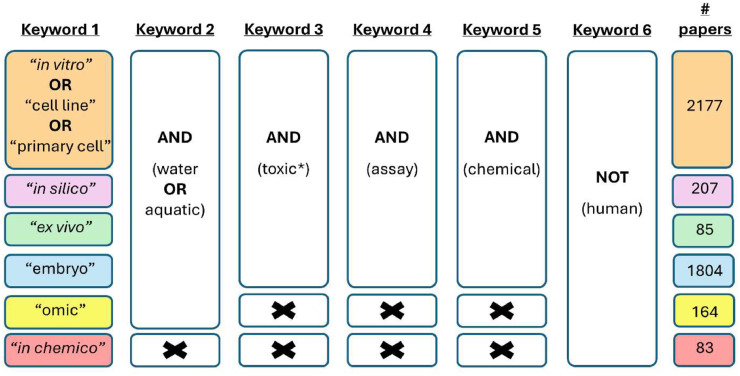
Search term strategy performed in PubMed to collect literature and number of retrieved papers. The asterisk (*) after the word toxic was used as a truncation symbol to search for all terms that begun with the same root.

A systematic search on PubMed was performed based on predefined keywords that encompass the full spectrum of NAMs and aimed at discovering papers utilizing assays for aquatic toxicity testing (Fig. 1). Since the focus was only on aquatic toxicity testing, articles on human toxicity methods were excluded from all search term strategies (Fig. 1, Keyword 6).

**Fig. 2:**
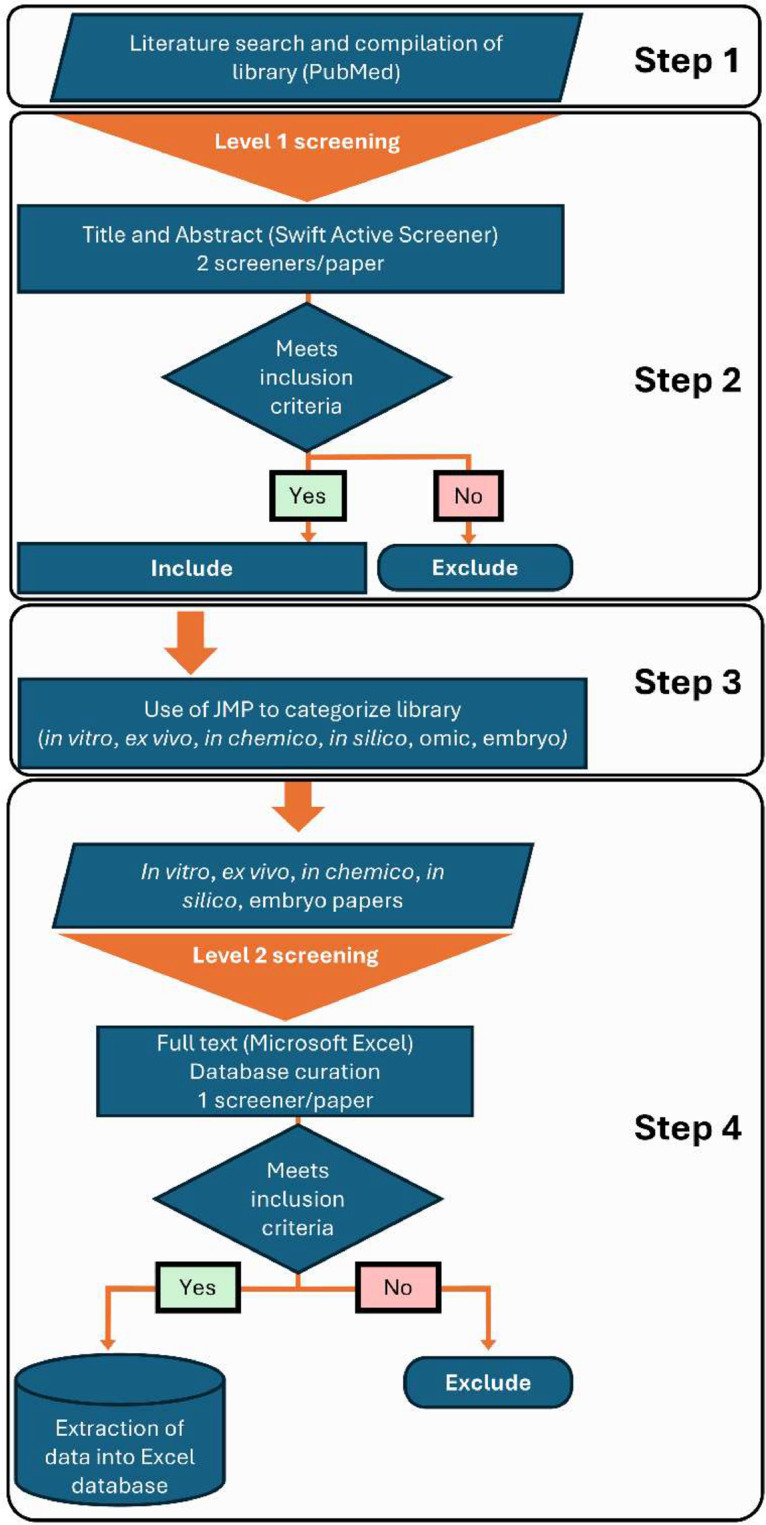
Summary of screening process for the systematic review of NAMs in aquatic toxicity testing.

The combination of keywords was not followed for each separate NAM since “*in chemico*” and “omic” papers were much less frequently retrieved than the other NAMs search terms. For each NAM a different search was performed, and a total of 4520 articles were retrieved for screening (Fig. 2, Step 1).

For Step 2, the systematic review tool SWIFT-Active Screener was used to screen the identified literature (Howard et al., 2020). Prior to screening, a library of all retrieved articles was created and duplicated articles were removed leaving 4275 articles for screening. This tool was selected because it uses machine learning to suggest the next articles to screen based on those previously included. The model is continuously refined according to the user's screening decisions. This literature prioritization process helps reduce both the number of articles that need to be screened and the overall screening time. Approximately 95% (inclusion threshold) of relevant articles are revealed after 40–50% of the library is screened.. Once this threshold is met, all unscreened articles are considered excluded. The Level 1 screening in SWIFT was performed on each article’s Title and Abstract by 2 screeners. Inclusion and exclusion criteria were agreed by the project team prior to the start of screening (Table 1). If an article was selected for inclusion or exclusion by both screeners, it was either included in the next steps of the review or not considered further. In cases of disagreement, the article was re-screened by both reviewers, who then reached a consensus on whether to include or exclude it from the subsequent stages (Fig. 1, Step 2).

**Table 1:** Step 2, Level 1 paper screening from wide literature search using SWIFT active screener (inclusion and exclusion criteria).

Inclusion	Exclusion
• *In vitro* exposure and assays (fish, amphibians and marine mammals); • Embryo exposure irrespective of species if the exposure doesn’t exceed the endogenous feeding stage; • Embryo exposures if they exceeded the non-protected life stage but only if they evaluated endpoints within the non-protected life stage period; • *In silico* approaches using *in vivo* data (both existing and experimental) if relevant species (*i.e.*, fish, amphibians, marine mammals); • Exposure of chemicals using microinjection; • Scientific papers written in English; • Fish receptor transfection or binding assays.	• *In vivo* exposures followed by *in vitro* assays; • *In vitro* studies with non-aquatic mammalian cells (*i.e.*, human, mice, rats); • Generation or offspring effect(s); • Early life stage/whole life cycle exposures; • Embryo papers when protected life stages were exposed to chemicals. • Yeast estrogen and androgen screening assays; • Studies using bacteria, algae, birds or invertebrates; • Reviews, commentaries or concept papers; • Book chapters;Restricted or subscription only scientific papers; • Methodological papers, i.e., characterization of cell lines, optimization of biological assays without further chemical exposure; • Embryo papers that investigated the effects of chemicals only on traditional toxicity endpoints such as survival and morphology (malformations).

The exclusion criteria were based on the reasoning that original research papers were to be screened for collecting and reporting assays and NAMs used to study the effects of chemicals.

### 2.2 ATEST database construction and curation

For Step 3, the computer software JMP (version 17.2) was used to assign tags to the papers that were included during the Level 1 screening. The software was programmed to search for keywords in the title and abstract for the different NAMs (*in vitro*, cell line, primary cell, *in silico, in chemico, ex vivo*, omic and embryo). Each paper was assigned one or more tags based on the presence of these keywords (Fig. 2, Step 3). Full text screening (Level 2 screening) was initiated with *in vitro, ex vivo, in chemico, in silico* and embryo papers. Omic papers were not considered as a different NAM but rather a method of investigating chemical toxicity, but they were screened nevertheless, and the information was registered when an omic method (*e.g.* transcriptomic, metabolomic, lipidomic etc.) was applied to investigate the chemical toxicity in either cell- or tissue-based experiments. In Step 4 during Level 2 screening, information from each paper was added to 18 columns of the Excel database (https://zenodo.org/records/19728632). These 18 columns were: Inclusion, Type of NAM, Taxa, Species, Sex, Life stage, Organ/tissue, Type of test system, Cell type, Cellosaurus ID, Commercial cell line (Y/N), Commercial Accession number, Source, Cell line name, Type of method/technique, Method name, Endpoint (target) and Endpoint categorisation. Not all columns were applicable for the embryo screening, so the columns were reduced to Inclusion, Type of NAM, Taxa, Species, Organ/Tissue, Type of method/technique, Method name, Endpoint (target) and Endpoint categorisation (Fig. 2, Step 4).

For each paper that was screened, multiple rows could be created. Each row represented one case, *i.e.*, a unique method such as species, organ/tissue, type of test system etc. when multiple testing strategies were included in one paper. For example, if one paper tested three different methods to evaluate cytotoxicity, then three rows (cases) were registered for that paper. Since the Level 1 screening was performed only on the Title and Abstract the same exclusion criteria (see Table 1) were applied to Level 2, full text screening which provided the full description of the methodological approach.

## 3 Review summary

**Fig. 3:**
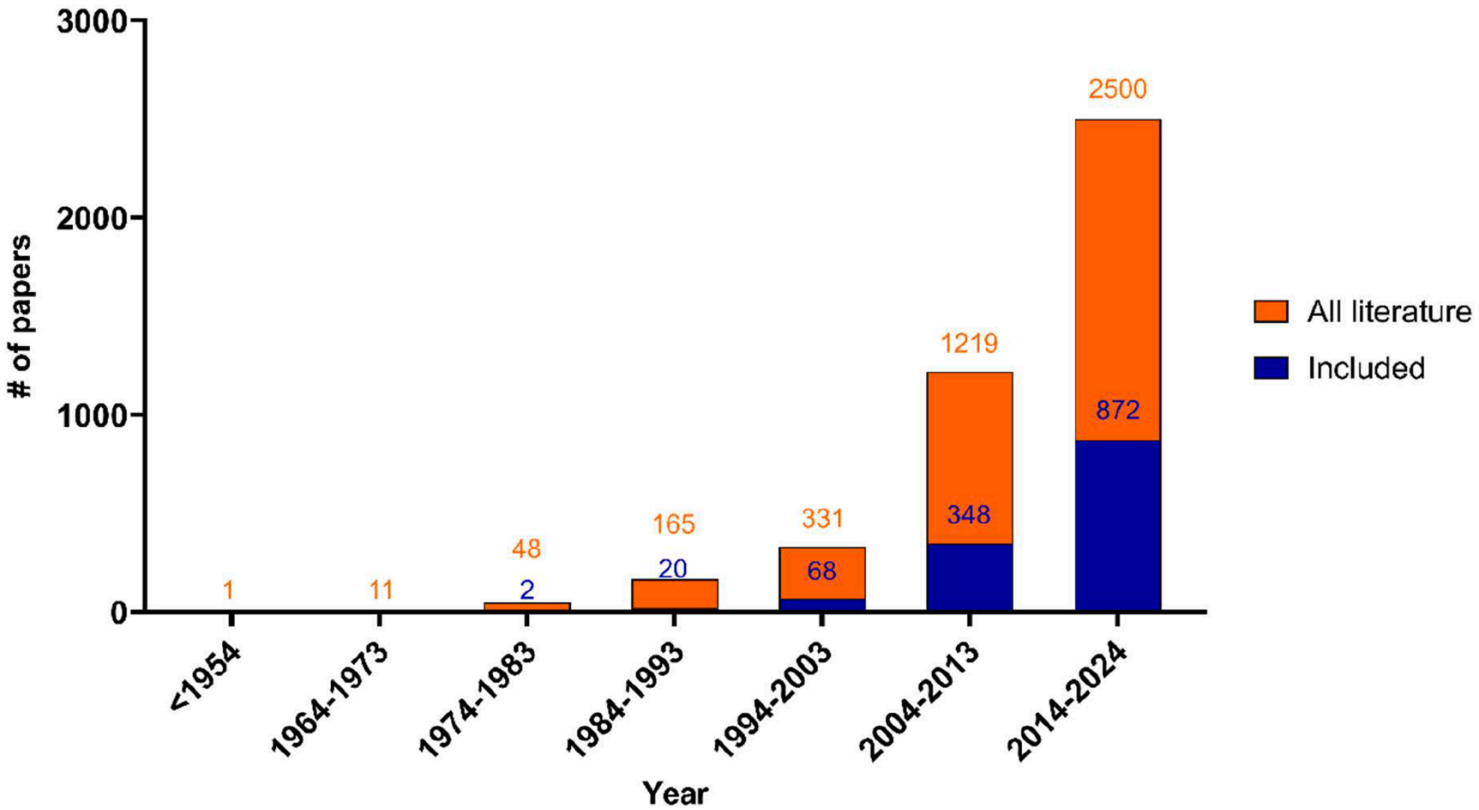
Count of papers that were captured during the literature search in PubMed (orange) and papers that were included after level 1 screening (blue).

Of the 4275 unique articles screened in Level 1, 1310 articles were selected for further Level 2 screening (Fig. 3). The data indicated a strong increasing trend for NAM papers over time with unsurprisingly most of the research being published over the last 10 years.

### 3.1 *In silico* approaches

**Fig. 4:**
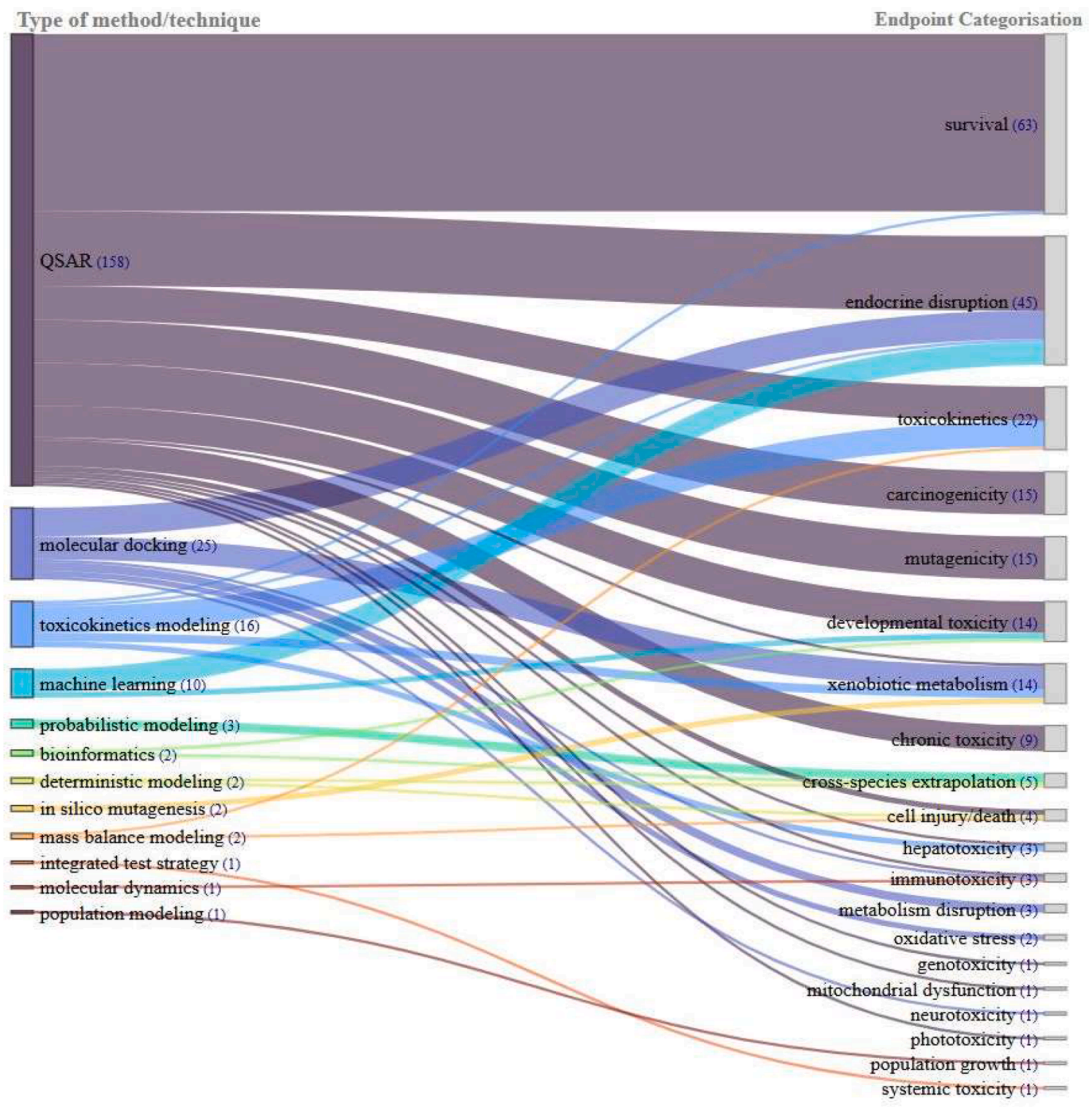
Sankey diagram depicting the overview of in silico NAMs (left hand side) and the respective endpoint categories covered by each in silico NAM (right hand side). The width of the lines is proportional to the number of cases (studies).

Based on the systematic review, a total of 223 cases were linked to the use of 80 unique *in silico* aquatic NAMs. These methods can be categorized into 12 groups, including QSAR, toxicokinetic modeling, probabilistic modeling, *in silico* mutagenesis, molecular docking, integrated test strategies, bioinformatics, machine learning, regression modeling, mass balance modeling, population modeling, molecular dynamics, and deterministic modeling (Fig. 4). These computational approaches addressed a wide range of endpoints, such as acute and chronic toxicity, receptor binding, bioaccumulation factor (BCF), biotransformation, and several mechanism-of-action (MoA)-specific effects, relevant across differing levels of biological organization (ATEST Database). While most cases focused on fish studies, some methods were also applicable to amphibians and reptiles. Importantly, most of the methods were not species-specific. While the majority of the *in silico* approaches reviewed are based on chemical structure or property, there are also specialized computational NAMs that focus on cross-scale (*e.g. in vitro* to *in vivo*) extrapolation of internal chemical concentrations, such as physiologically based toxicokinetic (PBTK) models, and cross-species extrapolation, such as the SeqAPASS and G2PScan tools.

### 3.2 *In chemico* assays

During the systematic review, five cases were identified that used *in chemico* testing. Four cases involved evaluating the effects of chemicals on acetylcholinesterase (AChE) derived from the electric organ of the electric eel (*Electrophorus electricus*). The remaining case focused on quantifying the effects on antioxidant levels, particularly on glutathione S-transferase (GST).

### 3.3 *In vitro* assays

**Fig. 5:**
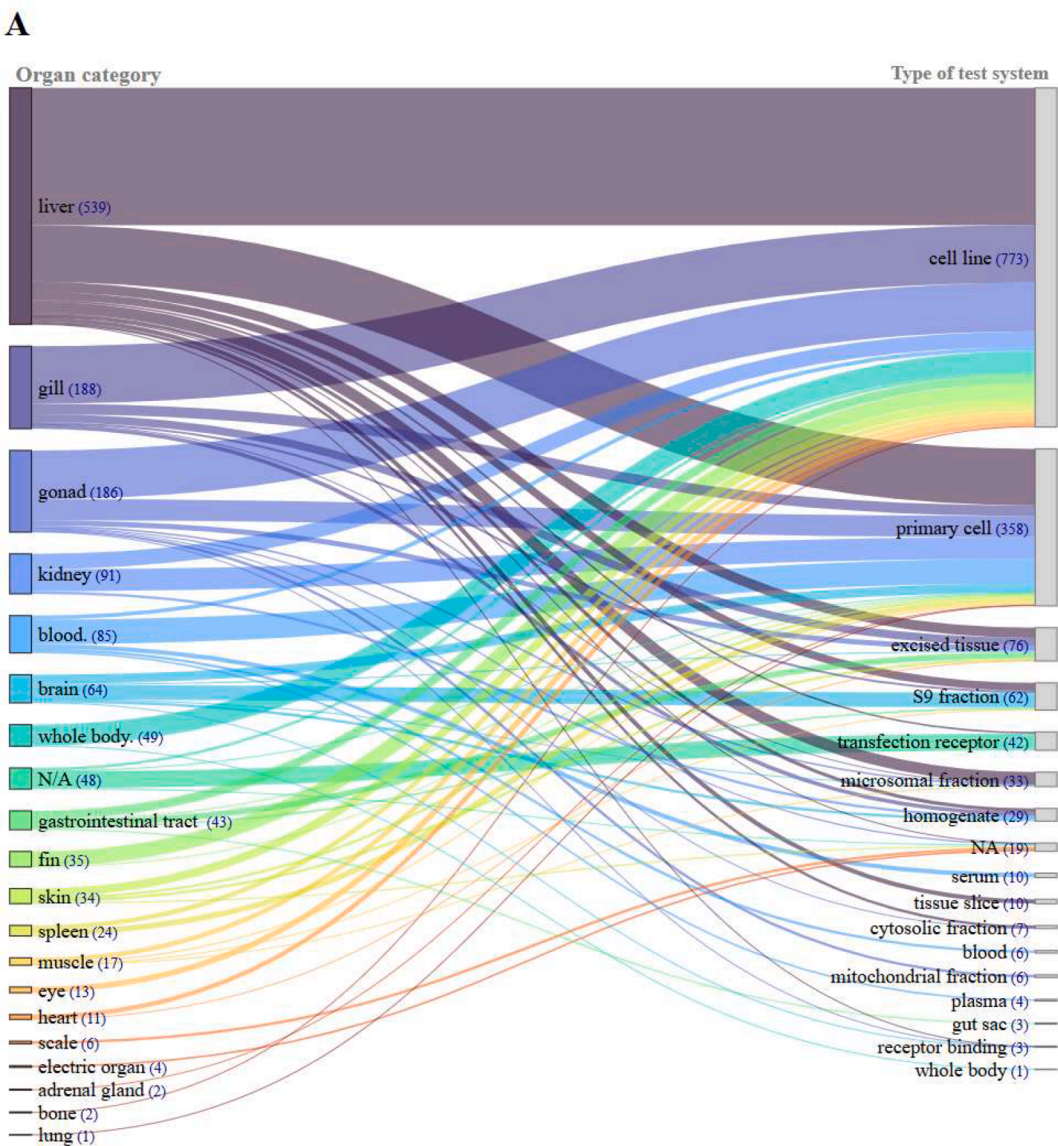

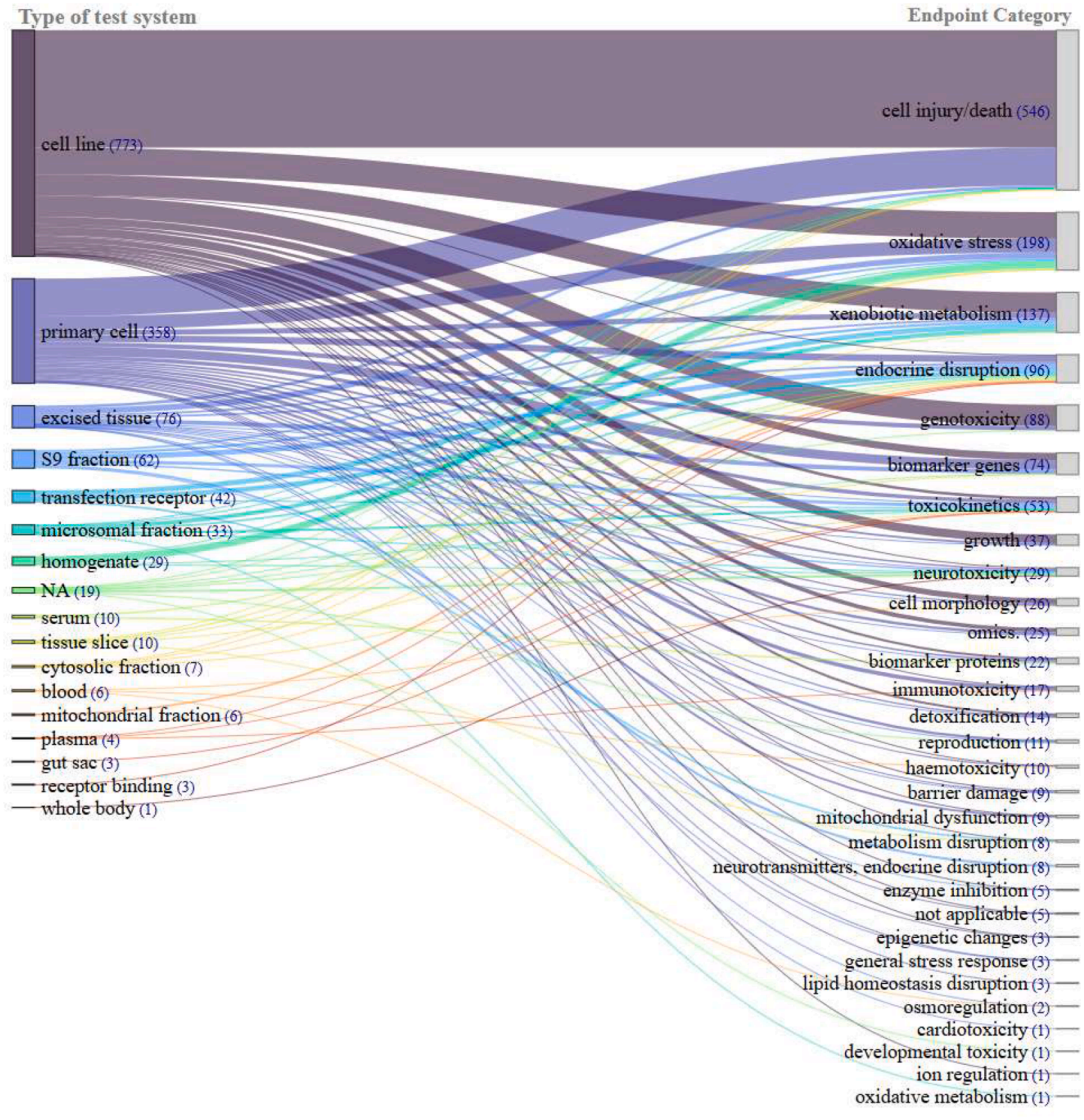
Sankey diagram of in chemico, *in vitro* and ex vivo NAMs. The top panel (A) represents the organ and type of test system while the bottom panel (B) represents the type of test system and endpoint category. The width of the lines is proportional to the number of cases (studies).

*In vitro* methods can provide mechanistic understanding of a chemical´s mode of action and support hazard assessment. The *in vitro* models identified in this review made use of cell lines (773 cases), followed by primary cells (358 cases), transfected cell lines, with various nuclear receptors (42 cases), and cell-free assays (137 cases incorporating analysis with homogenate, microsomal, cytosolic, S9 and mitochondrial fraction) (Fig. 5, Table 2, ATEST Database). In the reviewed literature, the most represented organ category was the liver (541 cases) followed by the gill (186 cases), gonad (130 cases), kidney (91 cases), brain (47 cases) and skin (34 cases). The most frequently reported assays for *in vitro* in the reviewed literature, reported baseline toxicity (cell injury/death), covering one or several endpoints such as cell membrane integrity, metabolic activity, lysosomal membrane integrity, LDH release, colony formation and ATP (Table 2). Other well-studied endpoint categories were oxidative stress, xenobiotic metabolism and endocrine disruption (for more detailed information see Table 2). Based on currently reviewed literature, several endpoint categories were poorly covered (<10 cases), such as cardiotoxicity, barrier damage, developmental toxicity, epigenetic changes, enzyme inhibition, general stress response, haemotoxicity, ion regulation, lipid homeostasis, metabolism disruption, mitochondrial dysfunction, neurotransmitters, osmoregulation and oxidative metabolism, highlighting the need for further development within these mechanisms of toxicity. A summarizing overview of the specific endpoints reviewed may be found in Table 2.

**Table 2:** Overview of reported endpoints studied in reviewed literature *in vitro* and *ex vivo* bioassays. Number of cases reported are specified in parentheses.

Endpoint category	Endpoint
Barrier damage (9)	Epithelial morphology and integrity (2), tight junction integrity (5), electrical impedance of a monolayer of endothelial cells (2)
Cell injury/death (546)	Metabolic activity (183), lysosomal membrane stability (116), Cell membrane integrity (98), Apoptosis (41), cell viability (40), DNA nuclei/content/presence (19), necrosis (15), cytotoxicity/protein content (10), energy metabolism (8), cellular death (7), cytochrome c (3), colony formation (5), cell cycle (3), cell proliferation (1), alpha oxidation in the peroxisome, ATP (1), cellular K+ (1), Ca2+ influx (1), tissue morphology (1), glycogen deposits (1), bioluminescence (1), lysosome presence (1), respiratory metabolism (1), mitochondrial membrane potential (1)
Cardiotoxicity (1)	Force and frequency of heart contractions (1)
Cell morphology (26)	Cell morphology (19), filamentous actin (3), cell structure changes (2), cell volume (1), optical appearance of fish chromatophores (1)
Detoxification (14)	Cell membrane efflux (ATP-binding) (6), metallothionein (5), ABCC activity (2), mdr1-like activity (1)
Developmental toxicity (1)	Sox7 (1)
Endocrine disruption (96)	Cytochrome P450 19 (Cyp19) (24), Estrogen receptor (19), Vitellogenin (13), Androgen receptor (7), protein binding: transthyretin or serum (5), steroid hormone level (11-Ketotestosterone, 17-β estradiol (E2), 17α,20β-Dihydroxy-4-pregnen-3-one) (4), corticosterone (4), E2-presence (3), cyp11b (2), cyp17 (2), alkaline phosphatase (ALP) (2), tartrate-resistant acid phosphatase (TRAP) (2), 20β-HSD activity (maturing inducing hormone) (2), vitamin D receptor (2), thyroid hormone receptor beta (TRβ) (1), growth-hormone-releasing hormone receptor (GhrhR) (1), gonadotropin hormone levels (1), Estrone metabolism (1)
Enzyme inhibition (5)	Digestive enzyme (3), succinate dehydrogenase activity (2)
Epigenetic changes (3)	DNA methylation (3)
General stress response (3)	Cortisol (1), goblet cell number (1), heat shock protein-70 (1)
Genotoxicity (88)	DNA damage (72), micronuclei formation (11), cell cycle (2), DNA double-strand breaks (1), DNA polymorphisms (1), DNA repair (1)
Growth (37)	Cell number (23), cell proliferation (9), adenylate cyclase (AC) (1), cyclic adenosine monophosphate (cAMP) (1), insulin-like growth factor 1 receptor (IGF-I) (1), proliferating cell nuclear antigen (PCNA) (1)
Haemotoxicity (10)	Haeme oxygenase 1 (HO-1)(2), haemoglobin (3), blood oxygen content (1), erythrocyte haemolysis (1), haemoglobin precipitation (1), haematocrit (1), hypoosmotic resistance (1)
Immunotoxicity (17)	Phagocytosis (8), lymphocyte cell proliferation (3), B-cell blastogenesis (2), lymphocyte blastogenesis (1) haemolysis (1), lysozyme activity (1), NK cell activity (1)
Ion regulation (1)	Quenching effect of metal ions: ca2+, pb2+ and cd2+ (1)
Lipid homeostasis disruption (3)	Cholesterol (1), membrane fluidity (1), triglyceride profile (1)
Metabolism disruption (8)	Peroxisome proliferator-activated receptor (6), free-thiol groups of de-acetylated coenzyme A (1), fatty acid profile (1)
Mitochondrial dysfunction (9)	Mitochondrial membrane potential (8), mitochondrial *Ca* (1)
Neurotoxicity (29)	Acetylcholinesterase (16), cholinesterase (12), ryanodine receptor 1 (RyR1) (1)
Neurotransmitters (8)	GABA(A) (2), GAD (2), dopamine-2 (D2) (1), glutamate (N-methyl-d-aspartic acid (NMDA) (1), muscarinic acetylcholine (mACh) (1), monoamine oxidase activity (MAO) (1)
Omics (25)	Transcriptomics (15), proteomics (4), metabolomics (3), Lipidomics (2), Lipid expression (1)
Osmoregulation (2)	Chloride cell number (1), haemolysis (1)
Oxidative metabolism (1)	Lipid-oxidative activity (1)
Oxidative stress (198)	Antioxidants (117): glutathione S-transferase (GST) (26), superoxide dismutase (SOD) (26), glutathione peroxidase (GPX) (17), catalase (14), glutathione reductase (GR) (14), glutathione (GSH) (10), glucose-6-phosphate dehydrogenase (4), nuclear factor erythroid 2-related factor 2 (3), cytochrome c reduction (1) total antioxidant capacity (TAC) (1); reactive oxygen species (ROS) (44), lipid peroxidation (LPO) (28), lipid hydroperoxides (2), carbonyl protein (2), 78-kDa glucose-regulated protein (GRP78) (1), ascorbic acid content (1), free radical scavenging capacity (1)
Reproduction (11)	Sperm quality (5), sperm motility (3), germinal vesicle breakdown (GVBD) in oocyte (1), oocyte maturation (1), Sperm morphology (1)
Toxicokinetics (53)	Biotransformation (33), bioaccumulation (10), uptake (8), distribution (2)
Xenobiotic metabolism (137)	Cyp1a (103), aryl hydrocarbon receptor (15), cyp2 (14), cyp3 (7), biotransformation (2), pregnane X-receptor (PXR) (2), aryl hydrocarbon nuclear translocator (1), constitutive active/androstane receptor (CAR) (1), cyp activities (unspecified) (1)
NA	Protein content (3), labelling of protein (2)

Note: Biomarker genes/proteins and omics were not included in the table.

Another approach that is steadily increasing is the application of various omics technologies to explore the effects of chemicals at the molecular level and uncover affected biological pathways. Primary cells and continuous cell lines originating from various organs were mainly used to study transcriptomics, followed by proteomics, metabolomics and lipidomics (ATEST database). Application of multi-omic technologies *in vitro* research has the potential to provide robust alternatives to traditional single endpoint testing by offering deeper and broader insights into mechanisms and enhancing adverse outcome prediction (Gant et al., 2023) when combined with *in silico* modeling. In the reviewed literature, only a few studies were identified (3 cases), thus demonstrating the need for further development within this promising field (ATEST database).

Novel methodology such as high-content imaging (cell painting) allows high throughput analysis using microscopy. This enables the investigation of chemical effects on cell morphology and function at a sub-cellular level through quantitative multiplex image analysis (Bray et al., 2016). In the current review, only one study reported use of high-throughput image-based cell profiling to assess the impacts on mitochondrial morphology in humpback whale HuWa_TERT_ and HF cell lines (Hosen et al., 2023). Although phenotypic screening, through high-throughput microscopy, is a very promising tool to study biological perturbations (Bray et al., 2016; Willis et al., 2020) and to be used in screening-level chemical assessments (Nyffeler et al., 2023) it has not yet been widely used in the field of ecotoxicology, and it is still under development.

*In vitro* models of varying complexity have been identified from 2D monolayer cultures, cultures in scaffolds and transwell inserts, three-dimensional spheroids and organoids and microphysiological lab-on-a chip models. Cell lines and primary cells, derived from gill and gut of rainbow trout, have been cultured in semipermeable membranes mimicking the epithelia and enable the possibility to study the transport of chemicals, and impacts on the epithelial integrity (Fuchylo et al., 2022; Georgantzopoulou et al., 2018; Minghetti and Schirmer, 2019; Schnell et al., 2015). Cells grown on semipermeable membranes develop a polarized epithelium (Minghetti and Schirmer, 2019) and gill cells can tolerate apical water allowing whole effluent toxicity testing to be performed (Georgantzopoulou et al., 2018; Schnell et al., 2015). 3D spheroid fish models, using primary liver cells from rainbow trout (Hultman et al., 2019) and other cell lines *e.g.* PLHC-1 (Rodd et al., 2017), ZFL (Park et al., 2022) and RTL-W1 (Lammel et al., 2019) have been developed to assess biotransformation, xenobiotic metabolism and effects after prolonged exposure to contaminants (Park et al., 2022). Such 3D *in vitro* models offer the advantages of developing cell-cell interactions, a microenvironment that better mimics *in vivo* tissues and are viable for longer time compared to monolayer cultures.

Fluidic chips have been developed incorporating the RTgill-W1 cell line for detection of water contaminants (Brennan et al., 2012; Glawdel et al., 2009), or epithelial RTgutGC/fibroblastic RTgutF (Drieschner et al., 2019) to increase physiological relevance by incorporating fluid flow and shear stress. Only three studies using fluidic chips were captured in this review. Such microphysiological systems offer the advantage of controlling the cell microenvironment including the nutrient flow, chemical concentration gradients, shear stress etc. which is closer to the *in vivo* situation (Hu et al., 2024). Different cell types, representing different organ/tissues, can be combined to mimic interactions between organs. Although at an early stage of development, with limited application in environmental toxicology, it has a great potential for the future.

Furthermore, the use of combined cell types and systems (*e.g.* gill cells co-cultured on semi-permeable membrane inserts above liver cells or 3D hepatic spheroids on wells below) could be considered a promising future improvement akin to being a pseudo fish with the aquatic exposure environment being on top of the gill inserts and the basolateral layer below being considered the blood plasma of the fish. Similarly, to mimic uptake of chemicals through the intestine, gut cells could be co-cultured on cell well inserts with complementary liver cells of 3D spheroids below. Such enterohepatic systems, based on the co-culture of RTgutGC and RTL-W1 rainbow trout cells lines, has been developed to investigate the interference of metallic nanoparticles with metal homeostasis (Minghetti and Schirmer, 2019) and could be further developed and modified to be used in physiological, toxicological and bioaccumulation studies.

Despite the high potential of cell-based *in vitro* methods as NAMs, reagents of animal origin are often needed as supplements in cell culture media to maintain healthy proliferating cells such as foetal bovine serum (FBS). FBS is an undefined complex mixture of multiple components and due to geographical and seasonal variations, issues on batch-to-batch variability have been raised that can contribute to lack of reproducible data. This has been acknowledged at the OECD in the Guidance Document on Good *In Vitro* Method Practices (OECD, 2018). Initial efforts have focused on the use of serum-free media with chemically defined components for human cell cultures such as the A549 cell line (Chary et al., 2022) among others and the RTgill-W1 cell line (Jozef et al., 2025). More systematic studies are needed to develop and validate animal origin-free media and assess their suitability for cell growth-promoting capabilities, long-term culture of different cell lines ensuring the maintenance of the cell morphology and functionality, their performance and response to chemicals.

### 3.4 *Ex vivo* assays

During the screening of scientific papers for the ATEST database construction 101 *ex vivo* cases were registered which predominantly involved fish species with some cases of marine mammal species. Fish species that were predominantly used in *ex vivo* testing were *Danio rerio* (zebrafish), *Pimephales promelas* (fathead minnow), *Oncorhynchus mykiss* (rainbow trout), and *Esox lucius* (northern pike). The most used types of test systems were excised tissue and tissue slices, targeting organs such as the liver, gills, gastrointestinal tract and reproductive organs (testes, ovary). Testing methods varied widely, including colorimetric and fluorescence approaches (*e.g.*, lactate dehydrogenase detection and antioxidant enzymes), radiolabelling for uptake, transport and bioaccumulation studies, molecular techniques like qPCR for analysing gene expression and immunoassays measuring protein expression levels. The endpoints that were targeted in the scientific papers, evaluated the effects of chemicals on oxidative stress, cell injury and death, endocrine disruption and xenobiotic metabolism, as well as the effect on targeted biomarkers (genes, proteins) (Fig. 5, Table 2, ATEST Database). *Ex vivo* NAMs have the advantage of addressing the complexity of multicellular tissues, preserving the organ´s architecture and cell-cell interaction which is normally not easy to incorporate *in vitro* systems thus providing a link between *in vivo* and *in vitro* studies. Everted and non-everted isolated intestinal segments (De Anna et al., 2022) and gut sacs (Khan et al., 2017; Klinck and Wood, 2011) from *O. mykiss* were used to study chemical intestinal transport processes, interaction with the ABCC transporters and impact on enzymatic activities. Excised gills from *Solea senegalensis*, combined with histopathological analysis, were used for the assessment of the impact of chemicals on the gills, however loss of viability and structural integrity of the gill after 4 h limits its application for more long-term exposures (Mieiro et al., 2019). Precision-cut tissue slices (PCTS) are *ex vivo* tissue explants with a defined thickness and can be prepared for all solid tissue types. Precision-cut liver slices have been suggested as a valuable method to overcome the limitations of isolated cells and continuous cell lines and has been applied to fish *e.g. Salmo salar* (Lemaire et al., 2011) and *O. mykiss* (Schmieder et al., 2000).

### 3.5 Embryo assays

Most studies, employing embryos for the evaluation of the effects of chemicals, have been performed with zebrafish (*Danio rerio*). This underscores the growing popularity of zebrafish as a model fish species in environmental research (Embry et al., 2010), especially after the addition of the FET in the OECD test guideline programme (OECD, 2013a), as well as in broader mammalian and human research contexts. While zebrafish are increasingly recognized for their utility in these areas, it is important to note that they are not the sole model fish used in environmental toxicology. Apart from zebrafish, embryo assays from some other fish species, that are more traditionally used in some geographical areas (Japan, North America), were highly represented in the database such as marine and freshwater medaka (*Oryzias melastigma* and *Oryzias latipes*), fathead minnow (*Pimephales promelas*), brown trout (*Salmo trutta*) and rainbow trout (*Oncorhynchus mykiss*). For the amphibian species, the most used species was the African clawed frog (*Xenopus laevis*) (ATEST database).

**Fig. 6:**
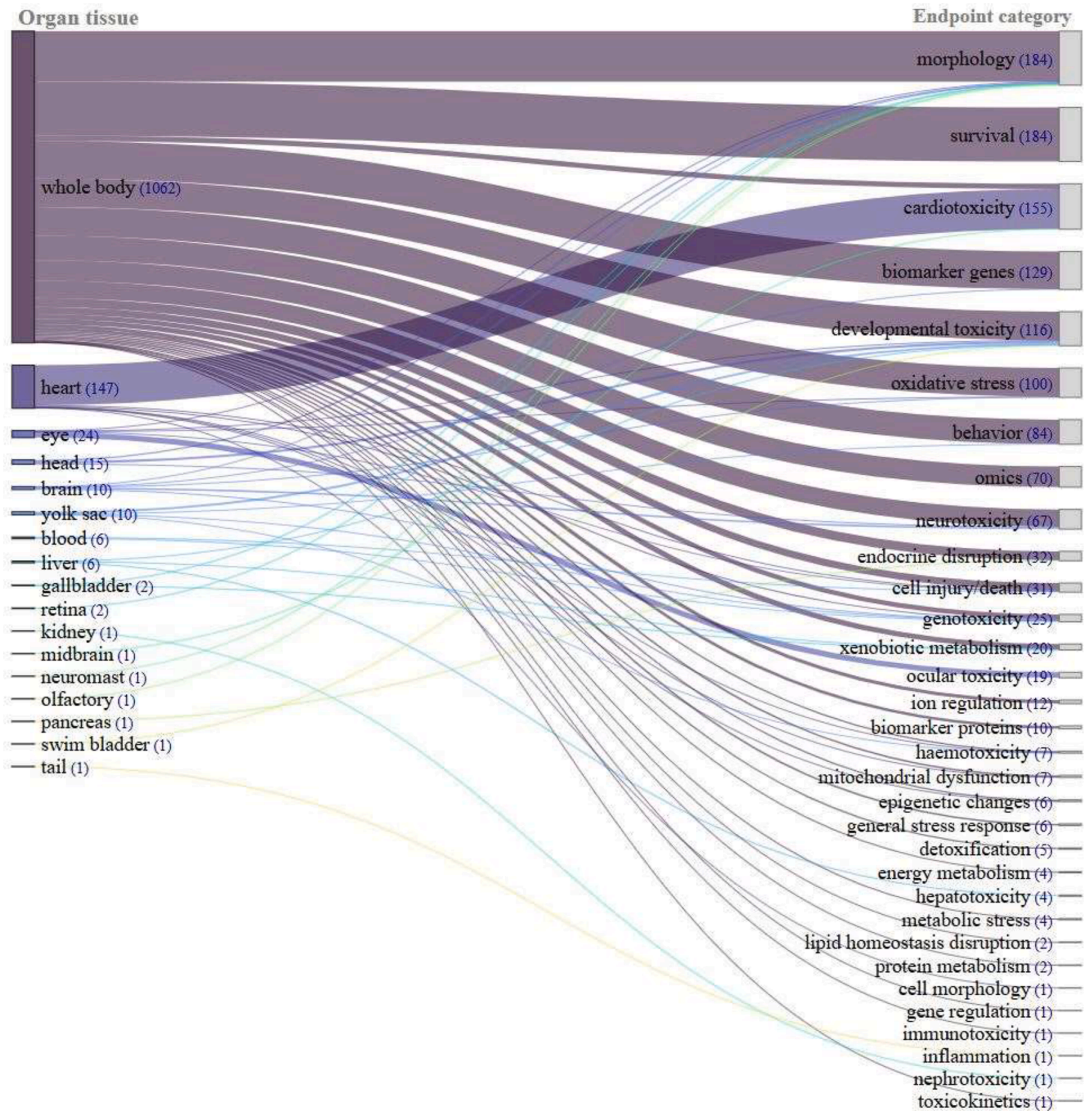
Sankey diagram illustrating the overview of embryo NAMs covering different organs/tissue and endpoint categories. The width of the lines is proportional to the number of cases (studies).

The study of the effects of chemicals on the morphology of embryos or eleutheroembryos (*i.e.*, malformation) and survival was reported in many articles, however further assays were used to distinguish other types of toxicity effects apart from general toxicity (Fig. 6). Morphometric measurements of distinct areas of embryos using machine learning algorithms (Dong et al., 2023; Teixidó et al., 2019) can provide further information not only on developmental arrest of different areas but link different phenotypes to different chemical classes. Ocular toxicity can easily be studied in embryos by measuring the eye area and morphology (Kim et al., 2019a, Kim et al., 2019b; Sørhus et al., 2023) as well as using histological sectioning to evaluate the effects on retinal morphology (Gaaied et al., 2022) and immunostaining to visualise Muller glial cells and photoreceptor cells (Chang et al., 2023). Additional endpoints that can provide further information on specific modes of toxicity, and specifically cardiotoxicity, that were predominantly used in the articles that were screened was the heart rate (Oliveira et al., 2017; Pruvot et al., 2012) as well as different aspects of the cardiovascular function such as cardiac output and contractility, blood flow velocity and stroke volume (Cypher et al., 2018; Li et al., 2019). An in-depth examination of heart and vascular development and morphology was conducted using different methods such as immunostaining (Jung et al., 2015; McIntyre et al., 2016), fluorescence microscopy and transgenic zebrafish lines (Chen et al., 2022; Kim et al., 2019a, Kim et al., 2019b). Behavioural tests such as the locomotor tests (Thomas et al., 2019), swimming activity (Jin et al., 2018), vision and touch evoke response assays (Madeira et al., 2018; Vasamsetti et al., 2021) and spontaneous or tail coiling assay (Fan et al., 2021; Ribeiro et al., 2022) can also provide an early indication of embryonic effects such as neurotoxicity, in a high-throughput manner due to the availability of automated tracking systems and software.

To further assess the effects of chemicals on neurotoxicological endpoints, apart from behavioural evaluation, many assays are available for embryos. The availability of multiple transgenic lines in zebrafish provides the opportunity to study effects in many structures such as the brain (Dzieciolowska et al., 2017), cerebellum (Wang et al., 2022), neurons and motor neurons (Gu et al., 2021; Kteeba et al., 2018). Immunostaining is another technique that provides opportunities for visualisation of the brain and neural network with the help of commercially available antibodies (de Soysa et al., 2012). Furthermore, different methods are available and have been utilised in the articles for the measurement of neurotransmitter levels such as dopamine, serotonin and gamma-aminobutyric acid, acetylcholinesterase (AChE) and cholinesterase (ChE) activities (Huang et al., 2021; Li et al., 2018; Santos et al., 2023; Stengel et al., 2018).

Markers of oxidative stress such as SOD, CAT, GST, GSH, LPO (Vieira et al., 2021), detoxification (Zhan et al., 2022), cell injury/death (acridine orange, caspases, TUNEL assay) (Di Paola et al., 2022; Jin et al., 2018), xenobiotic metabolism (EROD assay) (He et al., 2021) and genotoxicity (DNA damage, micronuclei formation) (Della Torre et al., 2018) are also utilized in embryo homogenates (Fig. 6). The potency of the toxic agent on elucidating effects on these markers, and a correlation of the response in each biological system, would be of interest for further investigations to eliminate the need for testing in multiple NAMs.

Some other approaches that have been employed in the articles that were screened at the genetic level are the gene knockdown using morpholinos (Teraoka et al., 2014) and *in situ* hybridization to explore localized gene expression (Ito et al., 2010). Both techniques are helpful in uncovering potential biomarkers and the function of individual genes after exposure to toxic substances.

Omics have previously demonstrated their ability to extract comprehensive biological insights by integrating large-scale molecular data. Multiple cases were registered in the database that have made use of one or more types of omics to explore the mode of toxicity (Chang et al., 2023; Lee et al., 2021). Points of departure, estimated through benchmark dose modeling of omics data, have also been utilized to determine response thresholds (Alcaraz et al., 2022, 2021).

Chemical degradation and metabolism during exposure experiments are factors hindering the evaluation of effects under a continuous exposure regime. Semi-static exposure with daily renewal of a percentage of exposure media is one solution in maintaining stable concentrations however handling stress might affect the outcome of the study. Zhu et al. (2015) developed a chip-based perfusion FET test that is performed in well plates and can maintain stable concentrations through constant flow rate. Their method also allows for the automatic acquisition of individual images from each well for the evaluation of morphometric effects (Zhu et al., 2015).

Some of the endpoints were not highly represented in the database. If this is due to lack of method availability or scientific interest remains to be determined. In certain areas however, such as research on the effects of toxic substances on mitochondrial function, high throughput methods are available. For example, immunofluorescent probes (Wirbisky et al., 2016) and instruments such as the Seahorse Extracellular Flux Analyzer (Agilent Technologies) enable efficient measurement of endpoints such as oxygen consumption rate and cellular respiration which are directly related to mitochondrial function (Lindberg and Di Giulio, 2019). A non-exhaustive list of other endpoints that require further use and development are epigenetic changes, hepatotoxicity and immunotoxicity (Fig. 6, Embryo ATEST database). Epigenetic changes through DNA methylation can affect the gene expression levels in exposed individuals but also be inherited in the next generations (Kamstra et al., 2017). Hepatotoxicity has been addressed in the screened papers but only in terms of liver development and morphology (Annunziato et al., 2020; Zhang et al., 2017). Since the liver is the main detoxification organ and is already developed and functional in embryonic stages (by 5 days post fertilization in zebrafish (Goessling and Sadler, 2015)), further biochemical assays should be developed and implemented in embryos for assessing toxicity. Immunotoxicity is included in the OECD 400 test guideline series dealing with testing methods to identify and characterize potential hazards in human health (OECD, 2023a). However, in environmental testing no such methods have been developed, evaluated and validated even when the immune system can play a crucial role in the defense of an organism against external and internal stressors. Only one case that the review identified investigated effects on the immune system by examining the macrophage accumulation of embryos exposed to metals and fluoroquinolones (Jia et al., 2022).

In summary, traditional toxicity endpoints such as survival and malformations are needed to establish sublethal concentrations, but they do not provide much mechanistic understanding of the effects of toxic substances. Additional endpoints that can be added to the FET test can provide further information on the toxicity and do not require the use of more animals. Methods such as measuring the heart rate and behavior outcome can provide evidence of potential cardiotoxic or neurotoxic effects. Moreover, if the mode of action of a substance, or the effect of a mixture is unknown, then the employment of omics is an ideal starting point to uncover mechanisms of toxicity and affected pathways and determine points of departure. These two types of NAMs can provide understanding of both concentrations that affect molecular events and affected pathways, while minimizing the use of animals and maximizing the magnitude of information. Understudied endpoints such as immunotoxicity, hepatotoxicity and epigenetic changes could be further developed and validated since the function of these three processes is pivotal for the response of an organism to toxic exposures. However, to further advance animal-free safety assessments, it should be considered that not all regions and stakeholders consider generating new embryo tests data as part of the NAM toolbox.

## 4 Current status and future strategies

### 4.1 Gap analysis

**Fig. 7:**
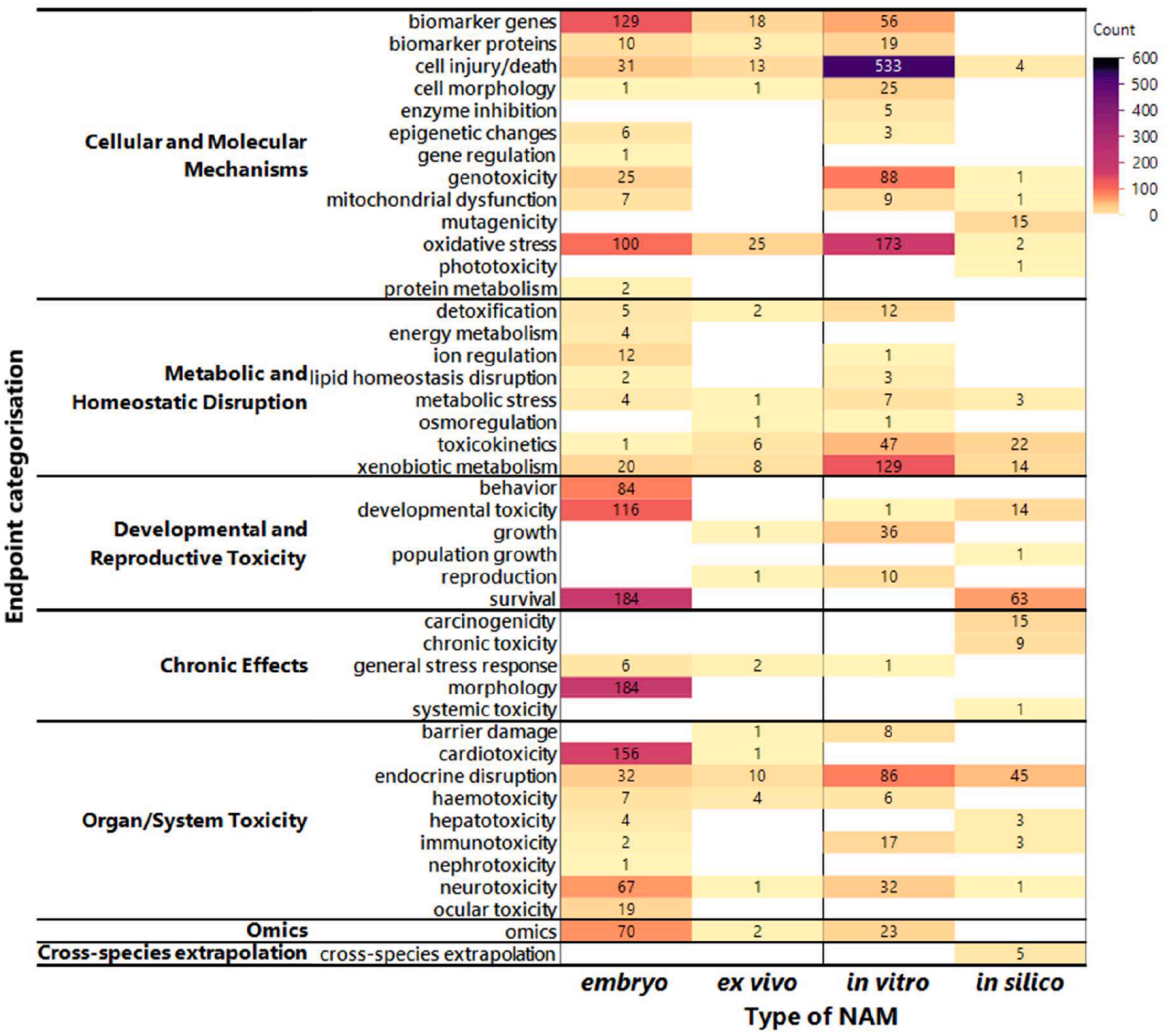
Gap analysis for the different NAMs reviewed in this study. Numbers in boxes are cases registered in the database. Organ toxicity captured *in vitro* such as hepatotoxicity and nephrotoxicity using liver and kidney cells have been registered under general cytotoxicity since the assays used capture cell injury or death. *In chemico* papers are excluded from this figure due to the low number of papers retrieved in the review.

The gap analysis with the JMP software after the screening process summarised the type of test system (for *in vitro* and *ex vivo*) that are used to evaluate the effect of the substances in the different endpoint categories, while for the *in silico* cases, the type of method/technique that was used (Fig. 7). Several of the endpoints are beyond traditional regulatory toxicity test requirements, which expand both the potential scope of the risk assessment as well as the technical rigor. However, challenges in application of these endpoints include potential lack of a validation dataset or demonstration of impact at ecologically relevant orders of complexity.

Based on the gap analysis, endpoints and methods, or test system for each NAM that require further development can be identified (Fig. 7). *In silico* methods, such as molecular dynamics, probabilistic risk assessment model and population modelling require further development. Methods that evaluate the effects of substances are also lacking for endpoints such as mitochondrial dysfunction, neurotoxicity, genotoxicity, phototoxicity and eight more endpoints where the registered cases are less than five from a total of 223 cases.

The gap analysis (Fig. 7) revealed a broad overlap among endpoint categories across *in vitro, ex vivo*, and embryo models. However, only a few endpoint categories—such as cell injury/death, endocrine disruption, oxidative stress, xenobiotic metabolism, toxicokinetics, and neurotoxicity—were fully covered by all reviewed NAMs. Most NAMs were only partially covered (missing one NAM) in categories like immunotoxicity, haemotoxicity, mitochondrial dysfunction, detoxification, cell morphology, general stress response, genotoxicity, and metabolism disruption. The analysis also highlights significant data gaps, particularly *in silico* and *ex vivo* approaches, due to a lack of reported cases.

Developments and further research are needed for the evaluation of chronic toxicity using *in vitro* models. As discussed in Section 3.3 the establishment and validation of cell models forming polarised epithelia in semipermeable membranes and spheroid/organoid *in vitro* and microfluidic organ-on-chip models can provide promising tools for chronic toxicity evaluation, and incorporation of more advanced co-culture systems can increase the relevance of existing *in vitro* models.

Further investigation (ATEST Database) revealed that several endpoint categories, such as epigenetic changes, remain underdeveloped despite their potential to predict both acute effects and long-term changes at low exposure concentrations. Integrating such analytical methods into *in vitro* systems could enhance the application of this NAM, especially when combined with modelling, to predict low-dose chronic toxicity.

Several of the endpoint categories are well-covered by one NAM, while not studied at all in others. Such an example is cardiotoxicity which has been extensively studied using embryo NAMs and an *ex vivo* model, pinpointing the need for further developments of heart *in vitro* models from different species as well as *in silico* models.

Other examples of poorly covered endpoint categories are immunotoxicity, where very few cases reported addressing this endpoint using *in silico, in vitro* or embryo assays. This is due to the lack of available cell models and the complex interplay between different organs, cell types and signalling molecules making it difficult to study the immune responses. A better understanding on the processes involved by the development of relevant NAM models and assays and applying more complex methodology, such as co-culturing and/or micro-fluid organ-on-chip models, are needed.

### 4.2 NAM readiness levels

There are many different methods available in the literature to critically evaluate NAMs and provide guidance on their use (Parish et al., 2020; van der Zalm et al., 2022). However, the first step that must be followed when evaluating the utility or “readiness” or any approach is to determine the specific context-of use for that method. The criteria against which any NAM must be evaluated is largely dependent on its intended use (*e.g.* prioritization, hazard screening, or risk assessment). For example, a NAM may be able to be utilized for priority setting even if a full laboratory ring trial has not been completed; whereas if that approach was going to be applied for classification or risk assessment, a more robust interlaboratory ring trial and/or full validation process would be required. It should be noted however, that at a minimum, any NAM requires an understanding of the method’s domain of applicability, its limitations, the data needs to be transparent and independently reviewed and be standardised and reproducible (van der Zalm et al., 2022). The level of development of any NAM can be considered by how ready the method is to be used or incorporated into regulatory frameworks (Table 3). For example, a completely novel NAM may be developed within a single research institute and may not have been trialled in different laboratories. This NAM would be at a very early stage of development and according to the proposed NAMs readiness criteria would be at a readiness level of 1 or 2 (*i.e.*, basic principles developed and investigated, and a standard operating procedure (SOP) being available. For any novel NAM development, it is imperative that a SOP is produced by the investigating laboratory and the method validated within the laboratory for multiple test compounds. In this situation, the method would be at a NAMs readiness level of 4 which would mean that the method is relatively robust but not sufficiently well tested to provide sufficient confidence in the method being used for regulatory purposes. To progress the NAM to a higher level of readiness (level 5 and 6), the method needs to be transferred to other laboratories in a coordinated ring trial. The ISO/Technical Specification 5594 (2022) describes guidance and requirements for designing an interlaboratory trial for validation of biotests and is recommended to be consulted by any NAM developers to ensure the minimum number of laboratories are included to provide a robust validation of the method. Only when a NAM has undergone successful and reproducibly rigorous interlaboratory ring trials should a NAM be considered mature enough to undergo the process of standardisation at an organisation such as the OECD or the ISO. This would then be at a NAMs readiness level of 7 or 8 according to the recommended scheme below. The importance of standardising methods is that data generated using such methods should be accepted under the mutual acceptance of data system to avoid duplication. The number of environmental NAMs available at higher readiness levels is fairly limited with very few reaching level 9 and inclusion in regulatory test requirements - *e.g.* for the EU Registration, Evaluation, Authorisation and Restriction of Chemicals (REACH) regulation the OECD 319 (hepatic biotransformation), the OECD 236 (FET test) and the OECD 249 (RTgill-W1 cytotoxicity assay) are at present level 8, and the OECD 321 (HYBIT) is at level 9. However, it should be noted that the development of a NAM may not need to reach a readiness level of 7 and above for it to be incorporated into hazard and risk assessments of chemicals since it may be very specialised with only a small number of laboratories capable of performing certain tests. In addition, geographical and political factors may mean that the NAMRL may not be the same in different regions. That said, it is recommended that any NAM method should be demonstrated to be transferable to other labs and the data produced from a lower level of readiness could still be useful on a weight of evidence basis or for prioritisation.

**Table 3:** Proposed levels of readiness for environmental new approach methodologies and reviewed literature covering proposed readiness levels.

NAM readiness level (NAMRL)	Description of the level of readiness	Examples of different methods at different levels of readiness from reviewed literature database (at the time of publishing this article)
NAMRL 1	Basic principles developed and investigated	Co-culturing *in vitro* test systems incorporating different cell types (Minghetti and Schirmer, 2019) High-content imaging (Hosen et al., 2023) Precision-cut tissue slices (PCTS) (Lemaire et al., 2011)
NAMRL 2	Experimental method formulated (standard operating procedure)	Multi-OMICS point of departure/moPOD (Song et al., 2023) G2PScan (Rivetti et al., 2023)
NAMRL 3	Experimental proof of concept	*Ex vivo* gut sacs (Khan et al., 2017; Klinck and Wood, 2011) Transactivation assays Transcriptomic point of departure/tPOD (O’Brien et al., 2025) SeqAPASS (LaLone et al., 2016)
NAMRL 4	Method validated in 1 laboratory (intra laboratory validation)	*In vitro* 3D hepatic spheroid intrinsic clearance rate assay (Hultman et al., 2019),
NAMRL 5	Method transferred successfully to multiple laboratories	Comet assay (*in vitro*)
NAMRL 6	Method validated in an interlaboratory ring trial	Behaviour assays for developmental neurotoxicity (zebrafish embryo using video tracking software for neurotoxicity) (currently under interlaboratory trial)
NAMRL 7	Method demonstrated to standardized organization (*e.g.* OECD, ISO)	Inclusion of thyroid endpoints in OECD fish tests
NAMRL 8	Method completed and approved by standardization organization	OECD 319 (hepatic biotransformation), FET test (OECD 236), RTgill-W1 cytotoxicity assay (OECD 249)^a^
NAMRL 9	Method accepted for regulatory test requirements (*e.g.* EU REACH)	EU REACH: *Hyallela azteca* bioconcentration test (HYBIT) OECD 321^b^

a These assays may advance to NAMRL 9 soon according to CARACAL recent meeting. Note that a NAM does not necessarily need to be at a RL of 9 as lower NAMRL can still be used for regulatory decision making within a WOE framework, but they just may not be used as a perfect 1:1 replacement.

b As of 16.10.2024 the member state committee accepted the HYBIT as a standard information requirement for REACH (All news - ECHA accessed 27.08.2025). Although invertebrate NAMs were out of scope in this review, HYBIT is included here because at the time of writing it is the only ecoNAM reaching level 9 and it is placed here as an example.

### 4.3 AOPs in supporting NAM applications

Although a wide range of biological endpoints has been addressed by current NAMs, their effective integration into regulatory decision-making, particularly for chemical hazard assessment, remains insufficiently defined. Many *in vitro* and *ex vivo* NAMs capture responses at lower levels of biological organization, such as molecular or cellular changes. However, regulatory decisions typically hinge on adverse outcomes at higher levels, such as impaired reproduction, growth inhibition, or population decline. Bridging this gap, in particular linking biomarker-type responses to ecologically relevant effects, remains a critical challenge. Adverse Outcome Pathways (AOPs) provide a structured solution to this problem. By organizing mechanistic knowledge into causal chains of events, starting with a molecular initiating event (MIE) and progressing through key events (KEs) to an adverse outcome (AO) of regulatory concern (Ankley et al., 2010), the AOP framework offers a scientifically robust means of contextualizing NAM data. This allows mechanistic data generated with *in vitro* or non-vertebrate models to be meaningfully interpreted in terms of impacts on wildlife health and population sustainability. As a central element of the Next Generation Risk Assessment (NGRA) toolbox, the AOP framework enables integration of diverse ecotoxicological data across multiple biological levels. They not only facilitate interpretation of individual NAM results but also provide a common framework to combine evidence from multiple sources, improving confidence in ecological hazard characterization and decision-making. For instance, to identify a chemical as an endocrine disruptor in the environment, information on endocrine activity of a chemical, which can be obtained by NAMs, needs to be linked to adverse effects at the population level. The link between the two relies on existing data organized in the form of AOPs, where NAM can support any events in the pathway. For example, *in vitro* assays for aromatase activity can detect chemicals that interfere with steroid hormone synthesis. An OECD endorsed AOP (AOPWiki, AOP #25, https://aopwiki.org/aops/25) linking aromatase inhibition (MIE) to reduced vitellogenin production (KE) and decreased fecundity (AO) in fish provides the mechanistic context to interpret the change of *in vitro* aromatase activity as an indicator of potential population-level reproductive impacts (Villeneuve, 2016).

In this review, NAM endpoints were mapped to proposed KEs in the AOP repository, AOP-Wiki (https://aopwiki.org/). Out of 1666 NAM endpoints (287 unique), 1316 (47 unique) were successfully linked to relevant KEs (*e.g.* KE 55 increase, cell injury/death, KE 1194 increase DNA damage, KE 1392, oxidative stress, KE 1521 decrease, growth). This demonstrates the feasibility and value of embedding NAM data within the AOP framework. At the same time, 240 unique NAM endpoints could not yet be mapped, underscoring gaps in current AOP coverage and highlighting the need for further development of new KEs and AOPs. The continued refinement and expansion of the AOP framework is therefore essential for realizing the full potential of NAMs in regulatory ecotoxicology as well as direct the identification and prioritisation of new NAM development. By enabling the translation of mechanistic data into outcomes of ecological concern, AOPs not only enhance the scientific credibility of NAMs but also strengthen the basis for environmental risk assessments that protect ecosystem health.

### 4.4 Integrated approaches to testing and assessment (IATA)

While the AOP framework is expected to play a key role in supporting the future regulatory application of NAMs, the current process for AOP development and regulatory endorsement is slow and requires substantial refinement. Furthermore, the framework itself needs significant further development. As the global shift toward phasing out animal testing accelerates, it becomes increasingly important to explore how existing NAMs can be more effectively utilized to inform and support regulatory decision-making. One promising approach is to leverage information from a variety of sources to facilitate a multiple-lines-of-evidence assessment. An Integrated Approach to Testing and Assessment (IATA) is a flexible framework that allows information from multiple methods and sources (*e.g., in silico, in vitro, in vivo*) to be combined and evaluated to inform a regulatory decision or guide additional data acquisition as defined by the specific context. The OECD has published several documents to help guide IATA development. The objective of the Integrated Approaches for Testing and Assessment (IATA) Case Studies Project is to increase experience with the use of IATA by developing case studies which constitute examples of predictions that are fit for regulatory use. The aim of this project is to create common understanding of using novel methodologies and the generation of considerations/guidance stemming from these case studies. Additionally, it serves as a mechanism to promote the development of IATAs for various endpoints and decision contexts, which are made publicly available. There are several IATAs that have been developed for ecological endpoints and include guidance for incorporation of NAM data, including a recent one on bioaccumulation that provides a robust and transparent framework to integrate lines of evidence including *in silico* and *in vitro* (*e.g.*, OECD 319) data (OECD 2024). Ideally, NAMs are evaluated and applied within a weight of evidence approach and integrated with existing and/or traditional approaches to support a decision. This may also be facilitated by incorporating defined approaches within future IATA developments.

### 4.5 OMICS as versatile NAMs

The development of high-content (HT) technologies such as next-generation sequencing has accelerated the rise of OMICS approaches and their consideration as versatile NAMs. OMICS investigate all molecules of a given type within a biological system (*e.g.*, genome, transcriptome, proteome, metabolome etc.), generating comprehensive datasets that capture system-wide biological responses. Unlike targeted NAM assays, which are typically designed for specific pathways, modes of action, or endpoints, OMICS assays offer a uniquely versatile platform. They can detect responses to chemicals with diverse and unknown modes of action and are applicable across different biological contexts, such as tissue types, developmental stages, sexes, or even across species. This breadth makes OMICS particularly powerful for addressing (eco)toxicological endpoints that are difficult or impossible to measure using targeted assays. In addition, OMICS technologies are highly flexible, ranging from targeted OMICS assays that focus on predefined sets of genes, proteins, or metabolites, to reduced-representation approaches that balance cost and coverage, to full OMICS profiling that captures the entire molecular landscape. Increasingly, multi-OMICS integration is also being applied, providing a holistic view of biological responses. This flexibility allows investigators to select the most appropriate balance between high-throughput (screening large chemical libraries) and high-content (in-depth mechanistic insight) depending on the testing needs. Traditionally, omics data have been used to uncover mechanisms of toxicity, identify biomarkers, and support AOP development by revealing molecular events that connect exposure to adverse outcomes.

Beyond mechanistic insights, OMICS can also provide quantitative data suitable for risk assessment. Molecular responses showing dose–response relationships can be analyzed using benchmark dose (BMD) modeling to derive OMICS-based points of departure (*e.g.*, transcriptomic POD [tPOD], metabolomic POD [mPOD] and multi-OMICS POD [moPOD]). Although a standard workflow for calculating tPODs is still under development, comparative studies provide guidance on best practices for both single OMICS assays (Pagé-Larivière et al., 2019; Villeneuve et al., 2024) and multi-OMICS (Song et al., 2023). Importantly, several studies have shown high concordance between OMICS-derived PODs and traditional apical PODs, particularly between acute tPODs and chronic aPODs (Costa et al., 2024; Pagé-Larivière et al., 2019). When supported by robust study design and analytical approaches, short-term OMICS studies can therefore provide protective exposure thresholds equivalent to chronic toxicity tests. Combined with public repositories (such as Gene Expression Omnibus (NIH, USA) and MetaboLights (EMBL-EBI, UK)), which facilitate data reuse and cross-validation, OMICS hold a strong potential as both versatile NAMs for broad biological coverage and quantitative tools for deriving points of departure in next-generation hazard and risk assessment (Haber et al., 2018).

### 4.6 Aligning aquatic toxicity and human safety assessments

In the OECD currently there are only two test guidelines for acute and *in vitro* aquatic toxicity testing, the TG 236 Fish Embryo Acute (FET) Test (OECD, 2013b), and the TG 249 Fish Cell Line Acute Toxicity (OECD, 2021b) based on the rainbow trout RTgill-W1 cell line. These two guidelines aim to test the effects of chemicals on the viability of the test system and provide effect or lethal concentrations for hazard assessment. However, tests that rely only on examining the effects of chemicals on survival, omit other important endpoints of toxicity that have been identified in this review such as genotoxicity, immunotoxicity and oxidative stress among others. Even though many cases of these endpoints have been registered in this review and laboratories have their own SOPs, published test guidelines are still lacking. In the interest of advancing and expanding testing for aquatic toxicity as to include additional endpoints, there are currently many test guidelines in the OECD Guidelines for the Testing of Chemicals, Section 4 that examine the effects of chemicals in these endpoints. A non-exhaustive list is the TG 444A: *in vitro* immunotoxicity: IL-2 Luc Assay (OECD, 2023a), TG 489: *in vivo* mammalian alkaline comet assay (OECD, 2016), TG 487: *in vitro* mammalian cell micronucleus test (OECD, 2023b) and TG 495: Ros (Reactive Oxygen Species) assay for photoreactivity (OECD 2019a, OECD, 2019b). These assays, even though they have been developed for mammalian cells, use the same principles or methods (*e.g.* comet assay, micronuclei assay) and can be used as the building blocks for the development of guidelines for these endpoints in aquatic toxicity testing.

## 5 Key recommendations from atest

• Standardisation and reproducibility assessment of NAMs is needed and the time taken from initiation to completion within international standardisation organisations (*e.g.* ISO and OECD) and regulatory frameworks (*e.g.* REACH) should be facilitated by multiple sources to ensure a smooth transition for inter laboratory validation of NAMs. It should be noted however, that NAMs may not always need to be at a high level of NAMs readiness (*e.g.* >6) to still be utilisable depending on their intended use, but they need to be fit for purpose (*e.g.* for prioritization purposes).
• Incorporation of multiple cell types/advanced co-culture models and complex 3D cell structures will increase physiological relevance of *in vitro* assays due to cell-cell interaction, multi-cell type (organ) communication and sequential cell type-cell type (organ-organ) chemical exposure. Further research is needed on the development of such models for chronic toxicity evaluation.
• Research should also be focused on developing chemically defined supplements as alternatives to fetal bovine serum (FBS) in cell culture media for different cell types.
• Endpoints of concern such as immunotoxicity, developmental and reproductive toxicity, cardiotoxicity, barrier damage, ion regulation, metabolism disruption, mitochondrial dysfunction, osmoregulation and oxidative metabolism are currently not well covered by NAMs, and assays are needed to be developed and evaluated.
• Further development of high content imaging (cell painting) will enable a more cost-efficient and high throughput analysis of chemical effects on cell morphology and function at a sub-cellular level.
• Toxicoepigenetics can be used as NAMs in reducing and possibly replacing chronic toxicity tests, as epigenomic changes may cause long lasting effects on the genome. Further efforts should therefore be considered to evaluate *in vitro* epigenomic changes with the aim to link it to adverse outcomes *in vivo*.
• To gain more insight of the effects of chemicals on early life stages of fish, sublethal endpoints could be included in fish embryo toxicity tests such as behavioural and/or endpoints covering endocrine disruption (*e.g.* thyroid as is currently under development at the OECD level). Development of different assays covering different endpoints are needed to bridge the gap between NAMs and *in vivo* endpoints currently not covered by the current NAMs.
• Regarding OMICS NAMS, critical needs for the future include:
  1) Advance OMICS as versatile NAMs capable of capturing system-wide biological responses across chemicals, modes of action, tissues, life stages, and species, especially for endpoints not measurable by targeted assays;
  2) Develop standardized workflows and best practices for deriving OMICS-based points of departure (*e.g.* tPODs, mPODs) to enable their consistent use in quantitative risk assessment. Data-sharing platforms need leveraging and multi-OMICS integration to enhance reproducibility, cross-validation, and regulatory acceptance of OMICS as next-generation tools in hazard and risk assessment.
• Future development of *in silico* NAMs should prioritize translational and read-across applications by advancing tools for cross-species extrapolation (*e.g.* amphibians, reptiles, and other underrepresented taxa) and cross-scale integration that links *in vitro* responses to population-level outcomes. Ecological relevance, predictive power and regulatory utility of *in silico* methods could be enhanced by expanding PBTK models; implementation of single gene/protein target (*e.g.* SeqAPASS) and biological pathway-level conservation analysis (*e.g.* G2PScan); and development of population modelling frameworks and QSARs capable of predicting chronic and sublethal endpoints.
• Future studies should explore the use of human NAM assays to screen for conserved mechanisms of toxicity relevant to aquatic organisms (*e.g.* genotoxicity, oxidative stress). Human NAM data could then be integrated as one line of evidence within IATA, alongside aquatic-specific data, to strengthen weight-of-evidence evaluations and accelerate the development of aquatic test guidelines for sublethal and mechanistic endpoints.
• AOP serves as a foundational organizing principle within NGRA, providing mechanistic coherence even when full pathway resolution is not required for decision-making. Future research should focus on expanding and refining the AOP framework, including the development of new KEs and pathways to capture NAM endpoints that are not yet represented. Studies are needed to strengthen the linkage between NAM outputs and AOPs, to better translate molecular and cellular responses into ecologically/regulatory relevant adverse outcomes. Further work should explore the use of AOPs as an integrative framework for combining diverse mechanistic datasets, thereby advancing next-generation risk assessment and improving ecological/regulatory relevance. In addition, AOP frameworks in cooperation with other NAMs should be aligned with defined approaches and integrated test strategies (or IATAs).

## CRediT authorship contribution statement

**Maria Christou:** Writing – review & editing, Writing – original draft, Visualization, Validation, Project administration, Methodology, Investigation, Formal analysis, Data curation. **Anastasia Georgantzopoulou:** Writing – review & editing, Writing – original draft, Visualization, Validation, Project administration, Methodology, Investigation, Funding acquisition, Formal analysis, Data curation, Conceptualization. **Maria Hultman:** Writing – review & editing, Writing – original draft, Investigation, Formal analysis, Data curation. **You Song:** Writing – review & editing, Writing – original draft, Methodology, Investigation, Funding acquisition, Formal analysis, Data curation. **Aina Charlotte Wennberg:** Writing – review & editing, Writing – original draft, Visualization, Software, Investigation, Data curation. **Sophie Mentzel:** Writing – review & editing, Writing – original draft, Visualization, Software. **Michelle Embry:** Writing – review & editing, Writing – original draft, Methodology, Investigation, Formal analysis, Data curation. **Amelie Ott:** Writing – review & editing, Project administration, Funding acquisition, Conceptualization. **Takahiro Suzuki:** Writing – review & editing. **Giacomo Grassi:** Writing – review & editing. **Kyle Roush:** Writing – review & editing. **Adam Lillicrap:** Writing – review & editing, Writing – original draft, Supervision, Resources, Project administration, Methodology, Investigation, Funding acquisition, Formal analysis, Data curation, Conceptualization.

## Declaration of competing interest

The authors declare that they do not have any competing interests

## Data Availability

Data will be made available on request.

## Appendix Supplementary materials

Image, application 1
